# Genetic variability of the *Avian leukosis* virus subgroup *J gp85* gene in layer flocks in Lower Egypt

**DOI:** 10.14202/vetworld.2020.1065-1072

**Published:** 2020-06-12

**Authors:** Nahed Yehia, Hemat S. El-Sayed, Sabry E. Omar, Fatma Amer

**Affiliations:** 1 Reference Laboratory for Veterinary Quality Control on Poultry Production, Animal Health Research Institute, Agriculture Research Center, Giza 12618, Egypt; 2 Department of Poultry Diseases, Benha Provincial Laboratory, Animal Health Research Institute, Agriculture Research Center, Giza 12618, Egypt

**Keywords:** *Avian leukosis(J)*, *gp85* gene, Marek’s disease, reticuloendotheliosis virus, tumor viruses

## Abstract

**Aim:**

This study aimed to determine the prevalence of layer flock tumor disease in Lower Egypt during the period of 2018-2019 and to undertake molecular characterization and determine the genetic diversity of all identified viruses.

**Materials and Methods:**

Forty samples were collected from layer chicken located in six governorates of Lower Egypt during the period of 2018-2019. Samples were taken from tumors in different organs. Tumor tissues were identified by histopathological sectioning and then further confirmed by a reverse-transcription polymerase chain reaction. Finally, genetic evolution of *Avian leukosis* virus (ALV-J) *gp85* gene was studied.

**Results:**

All the study samples were negative for Marek’s disease virus, reticuloendotheliosis virus, ALV (A,B,C and D) and 20 samples were positive for ALV-J in backyard in six governrates. Sequencing of ALV-J *gp85* gene was performed for six representative samples (one from each governorate), and they were found to be genetically related to prototype virus HPRS-1003 (identity percentage: 91.2-91.8%), but they were from a different group that was similar to the AF88-USA strain (first detected in 2000) with specific mutations, and they differed from a strain that was previously isolated in Egypt in 2005, forming two different subgroups (I and II) that had mutations in the hr1domain (V128F, R136A) and hr2 domain (S197G, E202K).

**Conclusion:**

The ALV-J virus was the main cause of neoplastic disease in layer chickens from Lower Egypt in the period of 2018-2019. We found that the genetic evolution of ALV-J *gp85* gene was related to prototype virus HPRS-1003 but in a different group with a specific mutation. Further studies are needed to evaluate the antigenicity and pathogenicity of recently detected ALV-J strains.

## Introduction

Marek’s disease virus (MDV), *Avian leukosis* virus (ALV), and reticuloendotheliosis virus (REV), collectively termed as avian tumor viruses, cause severe economic losses in the chicken industry [1].

MDV, which belongs to Alphaherpesvirinae subfamily, affects T-lymphocyte and causes nervous symptoms and ocular lesions [2]. REV, which belongs to the gammaretrovirus genus, causes bursal tumors by affecting pre-B and pre-T lymphocytes. [3].

ALV, which belongs to the Retroviridae family, genus Alpharetrovirus [4], is classified into 10 viral subgroups (A-I). The first six subgroups, (A-F), mainly infect chickens and turkeys, and they are classified according to viral envelope, host range, and cross-neutralization of the other uncommon subgroups (F, G, H, and I) of ALVs affected wild birds [5,6].

Chickens are most frequently infected by ALV subgroups A, B, and J. ALV-A causes lymphocytoma, hemangioma, and subcutaneous tumor in layer chicken [7], while ALV-B causes lymphocytic tumor and sarcomas [8]. ALV-C and ALV-D rarely affect chicken, and ALV-E has low pathogenicity in chicken [9].

The first detection of new group of ALV in the UK, 1980, and identified as ALV-J, then it was detected in broiler chicken in Great Britain in 1988 [4,5].

ALV-J was then detected sporadically in Japan in the early 1990s, but its incidence was reduced to negligible levels for a short period of time during which infected chickens were condemned, until infection levels increased again in 1998. ALV-J infection spreads rapidly outside of Japan, when it was detected in the USA, Taiwan, Israel, and a number of European countries. In the late 1990s, ALV-J spreads to Australia, and then, in early 2000, to China, Malaysia, and Egypt [10]. Numerous further cases of ALV-J were detected in both broiler and layer chickens in the period of 2000-2017 [11-14].

ALV is transmitted in chickens both vertically and horizontally. Control of vertical transmission can be achieved only by eliminating infected chickens [15,16].

The ALV-J causes both lymphoid leukosis and myeloid leukosis in poultry [17] as well as multiple tumors types that affecting liver, pancreas, kidney, ovary, mesenchyme, testis, and nervous system [5]. Importantly, there is not currently any vaccination or treatment available for ALV-J infection. Therefore, to prevent serious economic losses, it is critical to detect ALV-J infections early so that infected birds can be eliminated and further infection prevented.

The ALV genome, which consists of three structural proteins (gag/pro-pol/env), has been translated to the specific group antigen and envelops glycoprotein. Its genes are flanked by long terminal repeats that carry promoter and enhancer in the provirus form [18].

The *gp85* protein, a virus-encoded glycoprotein (*env*) gene, is the viral surface protein that primarily responsible for determining host range through viral entry into host cells, thus inducing host-neutralizing antibodies; it is also the major subgrouping determinant [19]. In this context, the *gp85* gene evolves rapidly when under host immune pressures the *gp85* gene evolving more rapidly in ALV-J compared to the ALV subgroups A-D [20] so that newly evolved ALV-J strains have been detected in many countries, causing further serious economic loss. Thus, it is necessary to continuously monitor the evolution of the *gp85* gene so that new strains and mutations that affect the pathogenesis of ALV can be detected.

Although virus isolation and tumor tissue histopathology have been routinely used for the differential diagnosis of avian oncogenic viruses, these methodologies are time-consuming and labor-intensive. Moreover, virus isolation is complicated when multiple infections are present. The histopathological examination is often difficult to identify lymphoid tumors lesions that are induced by different viruses [21]. Polymerase chain reaction (PCR) is currently the most accurate method of detecting many viral infections due to its technical advantages as a tool for differential diagnosis [3].

This study aims to identify the causative agent of tumor disease infection in flocks of chickens (layer flocks) in Lower Egypt during the period of 2018-2019 using molecular characterization and by studying genetic diversity within the detected virus.

## Materials and Methods

### Ethical approval

This study does not require the approval of the Institute Animal Ethics Committee.

### Sampling

Forty samples, which will be described in detail below, were collected from chickens in layer flocks that were located in the Lower Egypt region (El-Qalyubia, El-Monofia, El-Gharbia, EL-Behera, Alexandria, and El-Daqhlia) in the period of 2018-2019. The sampled chickens were recently diseased animals that had shown decreased levels of egg production and suffered from severe forms of tumor including hepatosplenomegaly, yellowish-white tumor on the visceral organs. The layer flocks were vaccinated against MDV using bivalent vaccine (HVT FC-126+Rispens CVI988), had 1% mortality. Samples were harvested from the following neoplastic organs; liver, spleen, heart, lung, brain, kidney, and nerves from freshly dead diseased birds. Samples were collected from three different chicken breeds: (i) Baladi (28 chickens), (ii) brown layers (6 chickens), and (iii) white layers (6 chickens). Chicken ages were in the range of 20-24 weeks.

The gross pathological lesions were identified at the time of necropsy.

After harvesting, the neoplastic organ samples were homogenized with 2000 IU/ml penicillin and 200 µg/ml streptomycin in saline. Homogenized tissues were then centrifuged for 15 min at 3000 rpm, and the resulting clear supernatant fluid was stored at −80°C until ready for examination.

### Histopathological examination

Liver and heart samples were fixed in 10% buffered formalin, then dehydrated in several grades of alcohol, embedded in paraffin, sectioned at 4µ thickness, and stained using hematoxylin and eosin stain described in Bancroft *et al*. [22].

### DNA extraction for PCR analysis

Since a sample types (liver, spleen, kidney, heart, and bursa of fabricius, proventriculus, brain, and nerve tissues) exhibited proliferative neoplastic changes, they underwent DNA extraction and PCR amplification. A mortar and pestle were used to grind (25 mg) before DNA extraction, which was performed using DNeasy Blood and tissue Kit (Qiagen, USA) according to the manufacturer’s protocol.

### Gene amplifications of MDV, ALV (A, B, C, D, and J), and REV

Viral DNA samples were amplified by gene-specific primers for MDV, ALV (A, B, C, D, and J), and REV using Phusion^®^ High-Fidelity DNA Polymerase (Thermo, MA, USA) and gene-specific primers (Table-1, [23-28]) according to the manufacturer’s protocol, at 98°C for 30 s and amplification for 40 cycles at a melting temperature of 98°C for 10 s, an annealing temperature according to each gene (Table-1) for 20 s and at an elongation temperature of 72°C for 1 min, and a final extension 72°C for 10 min. The gene-specific PCR amplicons were detected by agarose gel electrophoresis.

**Table-1:** Primers used for PCR amplification of tumor viruses.

Gene	Primer sequence	Annealing temp.	Fragment size	Reference
ALV A	H5-F GGATGAGGTGACTAAGAAAG EnvA- RAGAGAAAGAGGGGYGTCTAAGGAGA	48	694	[23]
ALV-B and D	BD-F CGAGAGTGGCTCGCGAGATGG BD-R AGCCGGACTATCGTATGGGGTAA	52	1100	[24]
ALV-C	C-F CGAGAGTGGCTCGCGAGATGG C-R CCCATATACCTCCTTTTCCTCTG	52	1400	[25]
ALV-J	H5-F GGATGAGGTGACTAAGAAAG H7-R CGAACCAAAGGTAACACACG	48	545	[26]
MDV	ICP4 F GGATCGCCCACCACGATTACTACC ICP4 R ACTGCC TCACACAACCTCATC TCC	58	318	[27]
REV	env-F AGCTAGGCTCGTATGAA env-R TATTGACCAGGTGGGTTG	48	438	[28]

PCR=Polymerase chain reaction, ALV=*Avian leukosis* virus

### The gp85 gene amplification and nucleotides sequence

Six positive samples were chosen to represent each of the six governorates and different breeds for *gp85* gene molecular characterization of ALV-J (Table-2). The following gene-specific primer**s** were designed for *gp85* gene amplification of ALV-J: (i) Forward primer, TTG GGA CCC CCA AGA ATT GG and (ii) reverse primer, AGAAGCAAATATCCGGGCTGT. Amplification was performed using Phusion^®^ High-Fidelity DNA Polymerase (Thermo, MA, USA) according to the manufacturer’s protocol at 98°C for 1 min and amplification for 40 cycles at a melting temperature of 98°C for 5 s, an annealing temperature of 48°C for 20 s and elongation temperature of 72°C for 1 min, and final extension at 72°C for 10 min. The gene-specific PCR amplicons were detected by agarose gel electrophoresis at 905 pb.

**Table-2:** Epidemiological data of selected sequenced strains.

Name of sample	Governorates	Date of collection	Breeds	Accession number
ALV-Egypt-QL1	El-Daqhlia	2-2109	Baladi	MN496121
ALV-Egypt-QL2	El-Monofia	11-2018	Brown	MN496122
ALV-Egypt-QL3	El-Gharbia	4-2018	Baladi	MN496123
ALV-Egypt-QL4	EL-Behera	5-2019	Baladi	MN496124
ALV-Egypt-QL5	Alexandria	12-2019	Baladi	MN496125
ALV-Egypt-QL6	El-Qalyubia	8-2018	White	MN496126

ALV=*Avian leukosis* virus

The positive amplicons were purified using the QIAquick Gel Extraction Kit (Qiagen, Hilden, Germany), and sequence reactions performed using the BigDye Terminator v3.1 Cycle Sequencing Kit (Applied Biosystems, California, USA) and using genes specific primers and the nucleotide sequence was determined using ABI 3500 Genetic Analyzer (Life Technologies, California, USA). All strains have been previously published by the National Center for Biotechnology Information.

### Genetic and phylogenetic analysis

Nucleotide and amino acid sequences were aligned with 20 related strains obtained from GenBank including the prototype ALV-J strain HPRS-103 which was used here as a reference strain [29], and specific strains that were frequently found in Egypt, USA, and China during the period of 2000-2017 using MegAlign module of DNASTAR software (Lasergene version 7.2; DNASTAR, Madison, WI, USA). Strains used in this study summarized in Supplementary Table-1. A phylogenetic tree was constructed using MEGA version 7 (www.megasoftware.net) by maximum likelihood tree method with moderate strength and 1000 bootstrap replicates [30]. The pair-wise nucleotide percent identity was calculated using DNASTAR Lasergene software (version 7.2; DNASTAR, Madison, WI, USA).

**Supplementary Table-1:** The data of the ALV-J reference strains.

Strains	Country	Accession number
ADOL-7501-2001	USA	AY027920.1
HPRS103 strain EgM/00-2005	Egypt	DQ316906.1
HPRS103 strain TgM/00-2005	Egypt	DQ316907.1
HPRS103 strain YSL02/00-2005	Egypt	DQ316908.1
GX14YYA1-2017	China	MF461280.1
GX14ZS14-2016	China	KX037423.1
UD4-2000	USA	AF307951.1
GD14J2-2016	China	KU500032
AF88-2000	USA	AF247390.1
6803-2000	USA	AF247388.1
BJ0301-2005	China	AY897230.1
6827-2000	USA	AF247389.1
1696-2000	USA	AAF66422.1
HUB09JY03-2010	China	AED99832.1
DBYJ1101-2013	China	AGS43001.1
DBYJ1105-2013	China	AGS42999.1
HPRS103-1994	USA	Z46390.1
SCSM00-2013	China	AHH25125.1
SDAU1704-2017	China	AVT42837.1

ALV=*Avian leukosis* virus

## Results

### Clinical signs

Before their death, the study chickens had suffered from emaciation, weakness, inappetence, dehydration, and reduced egg production with 1% mortality rate.

### Gross pathology

Postmortem lesions from freshly dead chickens had the following pathologies: (i) Diffuse lymphoid tumors in the liver with small nodules of diameter <1 mm and (ii) enlarged spleen, mesentery, and kidneys of up to 5 times the size of healthy organs. Tumors were smooth and soft, with diameters in the range of 2-5 mm. No enlargement of peripheral nerves was detected.

### Histopathological examination

**Figure-1:**
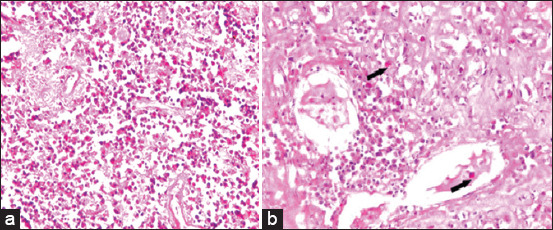
Histopathological lesion of liver. FN: a: The figure shows liver of chickens showed necrotic and severe degenerative changes of hepatocytes with massive myelocytic infiltrations in between the hepatocytes mixed with some lymphocytes (hematoxylin and eosin ×600). b: The figure shows liver of chickens showed intravascular (arrow) and extravascular myelocytic infiltrations (arrow) between the degenerated hepatocytes (hematoxylin and eosin ×640).

**Figure-2:**
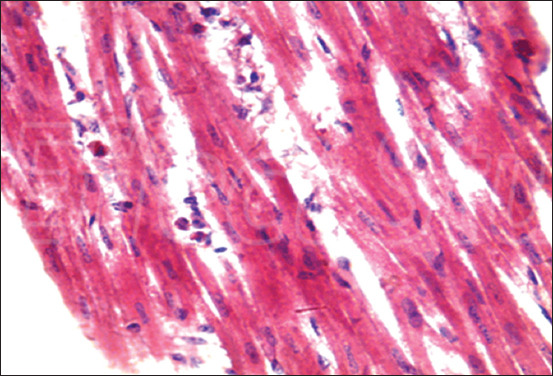
Histopathological lesion of affected heart. FN: The figure shows heart of chicken showed edema with infiltration of myelocytes (hematoxylin and eosin ×400).

Liver samples of chicken showed necrotic and severe degenerative changes to the hepatocytes with massive myelocytic infiltrations in between the hepatocytes mixed with some lymphocytes (Figure-1a) as well as intravascular and extravascular myelocytic infiltrations between the degenerated hepatocytes (Figure-1b). There were moderate edema and myelocytes infiltration in the myocardium (Figure-2).

### Polymerase chain reaction results

Of the total sample of 40 layer chickens, 20 samples were found to be infected by ALV-J at the 545 pb env gene; these chickens came from all three layer breeds (15 Baladi, two brown layers, and three white layers), as described in Table-3, and no cases of ALV (A, B, C, and D), MDV, or REV were found in these governorates.

**Table-3:** The result of PCR in layer flocks in different governorates.

Governorates	Number of tested flocks	Number of positive sample for ALV-J	Breeds
El-Qalyubia	20	10	15 Baladi, 5 brown
El-Monofia	5	2	1 brown, 4 white
El-Gharbia	3	3	3 Baladi
EL-Behera	5	2	5 Baladi
Alexandria	4	2	4 Baladi
El-Daqhlia	3	1	1 Baladi, 2 white

PCR=Polymerase chain reaction

### Molecular characterization of the ALV-J gp85 gene

**Figure-3:**
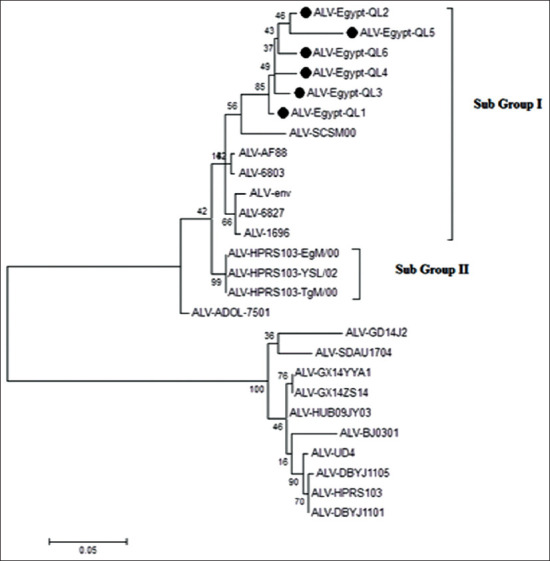
Phylogenetic tree of *gp85* gene of *Avian leukosis* virus (ALV) (J). FN: The figure shows the phylogenetic analysis of *gp85* gene of ALV-J gene reveling that all Egyptian strains cluster in the same group with two minor subgroups (I and II). The ALV(J) viruses in our study are indicated with a black dot.

PCR analysis of samples from ALV-J-infected chickens revealed that the *gp85* gene was positive PCR at 717pb. Phylogenetic analysis indicated that the Egyptian ALV-J strains were genetically related to prototype virus HPRS-1003 with identity percentage range 91.2-91.8%, but they were in a different group similar to AF88, ADOL-7501, and SCSM00-2013, all of which were found in chicken flocks located in the USA in the period of 2000-2001 and in China in the year 2013, respectively. It was published in GenBank accession number (MN496121-MN496126). When compared with prototype strain HPRS103, the sequence analysis in our study classifies the Egyptian virus strains into two different minor subgroups (I and II), as shown in Figure-3.

Subgroup I was found to have 23 amino acid mutations (R20Q, I40V, I42L, Q44S, P46Q, N48E, T49T, K51R, V54T, T55V, V57Y, A59G, D61K, N63D, K75Q, A76S, T79R, V128F, R136A, S197G, E202K, E240K, and H304R). While, the subgroup II had had 27 amino acid mutations (R20Q, I40V, I42L, Q44S, P46Q, N48E, T49T, K51R, V54T, T55V, V57H, A59G, D61K, N63D, T64S, L66T, S68T, K75Q, A76S, T79H, Q94R, V128F, R136A, S197G, E202K, E240K, and H304R).

The Egyptian strain identified in the study had several interesting specific features. In particular, it had L66A and S68T, while the prototype strain HPRS-103 did not. Furthermore, the QL6 of the Egyptian strains had the following novel and previously unpublished characteristics: A59E, S68A, and QL2 in K75R and S68A and QL3 in L66T.

Although there were no changes to the vr2 domain of the *gp85* gene in the study strains, there were two mutations (V128F and R136A) in the hr1 domain and other two (S197G and E202K) in the hr2 domain.

**Figure-4:**
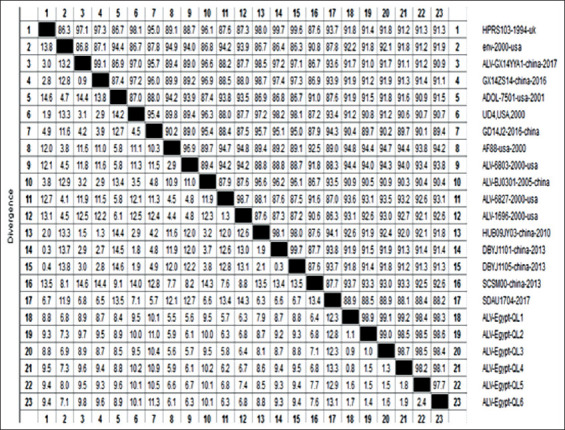
Nucleotide identities and divergence of sequenced viruses compared to other selected strains from China and the USA. FN: The figure shows comparative alignment of *gp85* gene showed that *gp85* nucleotide identity percent of all Egyptian strains in our study ranging from 88 to 94% when compared with different reference strains.

The nucleotide identity percentage of the study strains was found to be in the range of 88-94% when compared to reference strains found in the USA and China in the year 2000 and 2013, respectively. Furthermore, the nucleotide identity percentage of the study strains was found to be in the range of 91.2-91.8% when compared to the prototype strain HPRS103, in the range of 93.8-94.8 when compared to the AF88-2000, USA, and in the range of 90.9-91.9% when compared to the ADOL-7501-2001, USA, as shown in Figure-4.

## Discussion

Tumors are a leading cause of poultry deaths, livestock condemnation, and immune suppression induced by tumor viruses, leading to economic losses in the global poultry industry including that of Egypt, determination of the main causes of neoplasia in poultry remains challenging, and no vaccines are currently available, despite significant research. The most important neoplastic diseases that hinder the poultry industry include ALV, REV, and MDV, all of which are caused by retroviruses and herpesviruses [18].

Marek’s disease results in neoplastic tumors and immunosuppression due to T-lymphocytes involvements [31,32], while REV results in lymphoma of the bursa and T cells due to pre-T and B lymphocytes involvements [33]. ALV is classified into several subgroups (A, B, C, D, E, and J) based on the viral envelope glycoprotein [34]. With the exception of ALV-E, which affects B-lymphocytes, causing B-cell lymphoma, all other ALV subgroups are exogenous. ALV-J transforms the Fabricius bursa and causes metastasis to other visceral organs [35].

ALVs are known to spread rapidly through poultry populations across the world and new strains have been found to originate in one country and then spread rapidly to other countries, being difficult to control through restriction methods [18]. Infection is difficult to control without vaccination so that the only available control method is condemnation of infected flocks; indeed, many countries, including the USA, have only managed to control infection by carefully selecting non-infected breeders for broiler and layer production [31].

In Egypt, the ALV-J virus was detected for the 1^st^ time in the year 2000, when it was found in broiler chickens similar to HPRS-103 strain, after which it quickly spread throughout Egyptian poultry flocks [10-14]. After 2014, the range of infected host species widened from broiler chickens to reach layer chicken and wild ducks, with high mortality rates observed across infected species [13,36].

This study sought to identify using histopathological and molecular methods, the cause of recent infection that leads to tumor diseases in layer flocks (Baladi, brown layers, and white layers) located in Lower Egypt during 2018-2019.

Forty samples were collected from chickens located in six governorates in Lower Egypt; the study chickens suffered from emaciation, weakness, inappetence, dehydration, and low egg production, and they had enlarged livers and bursa on palpation. Postmortem revealed diffuse tumor in liver, spleen, and kidney. Histopathological examination revealed the lesion to be typical of ALV-J degenerative changes to hepatocytes, with massive myelocytic infiltrations of hepatocytes and myocardium, as previously described [37].

As stated in Davidson [21], it is difficult to detect viral-induced lymphoid tumors using histopathological examination, with PCR being the most appropriate and rapid method to detect many of the neoplastic viruses that affect poultry production [3]. In this study, PCR was used to test samples for ALV (A, B, C, D, and J), MDV, and REV. ALV-J infection was found in 20 from a range of governorates (10 in El-Qalyubia, two in El-Monofia, three in El-Gharbia, two in EL-Behera, two in Alexandria, and one in El-Daqhlia), with the highest number of infected chickens found in El-Qalyubia. Our results indicated that ALV-J is the main cause of viral-related tumor infections in chicken in the Lower Egypt.

The ALV genome consists of three structural proteins, gag/pro-pol/env [18]. The ALV glycoprotein envelope, which includes *gp85*, is highly evolved, carrying the receptor-binding site and inducing the host-neutralizing antibodies; the receptor-binding site is responsible for viral entry into the specific cell that determines the host range [38].

Genetic variability and antigenic difference have been found with sequence modification in the ALV-J *gp85* gene [20,39]. To detect the genetic evolution of the *gp85* gene in our samples, it was sequenced and compared to the ALV-J sequence obtained from prototype strain HPRS-103, known Egyptian strains and other reference strains that were isolated in China and the USA in the period of 2000-2017. Identity percentage was in the range of 88-94%. Phylogenetic analysis reveals that the Egyptian strains in our study are genetically related to prototype virus HPRS-1003 (identity percentage; 91.2-91.8%), but they fall into a different group that is similar to strain AF88-USA which was first identified in 2000 [40].

In our study, the alignment of amino acid sequences with six-layer isolates revealed 24 amino acid substitutions distributed along with the *gp85* subunit compared to prototype strain HPRS-10; this finding is in agreement with data from most strain isolated in the USA [39]. This strain can be classified into new minor subgroups that differ from the previous one isolated in Egypt in 2005. Our findings might be best explained using immune selective pressure as previously described [41].

The previous studies on *gp85* gene indicated that it contains important five variable regions hr1, hr2, vr1, vr2, and vr3 [41]. The hr1, hr2, and vr3 regions are responsible for receptor interaction with the host cell [24,42]. The *gp85* gene is characterized as being highly evolved, such that its changed the antigenic properties are the result of immune pressure [39]. In the present study, no change was found in v2 and V128F and R136A in the hr1 domain and S197G and E202K in the hr2 domain similar to most strain isolated in the USA [38]. The result obtained in this study suggests that these amino acid substitutions might be associated with changes in the pathogenicity and host range of ALV-J. Further studies are needed to fully evaluate the antigenicity and pathogenicity of the recently detected ALV-J strains.

## Conclusion

We conclude that ALV-J infection was the main cause of neoplastic disease in layer chicken located in Lower Egypt during the period of 2018-2019, with the highest infection rate being found in EL-Qalyubia. We found that the evolution of the ALV-J *gp85* gene is related to prototype virus HPRS-1003 (identity percentage: 91.2-91.8%), but that belongs in a different group, similar to the AF88-USA strain with specific mutations in the hr1 and hr2 domains forming two different minor subgroups (I and II). Further studies are needed to further evaluate the antigenicity and pathogenicity of the recently detected ALV-J strains.

## Authors’ Contributions

HSE and SEO collected samples. NY and FA were molecular characterization of samples and histopathological examination. All authors were involved in the writing, analysis of the data, and reviewed the manuscript, and they approved the final manuscript.

## References

[1] Witter R, Schat K, Saif Y.M (2003). Marek's disease. Disease of Poultry. Iowa State Press.

[2] Abd-Ellatieff H.A, Abou-Rawash A.A, Ellakany H.F, Yanai T (2017a). Th1 and th2 cytokines activity during transformation and lymphoma formation stage in chicken naturally infected with Marek's disease virus. IOSR. J. Agric. Vet. Sci.

[3] Davidson I (2009). Diverse uses of feathers with emphasis on diagnosis of avian viral infections and vaccine virus monitoring. Braz. J. Vet. Sci.

[4] Payne L.N, Brown S.R, Bumstead N, Howes K, Frazier J.A, Thouless M.E (1991). A novel subgroup of exogenous avian leukosis virus in chickens. J. Gen. Virol.

[5] Payne L.N, Gillespie A.M, Howes K (1992). Myeloid leukaemogenicity and transmission of the HPRS-103 strain of avian leukosis virus. Leukemia.

[6] McNally M.M, Wahlin K.J, Canto-Soler M.V (2010). Endogenous expression of ASLV viral proteins in specific pathogen free chicken embryos:Relevance for the developmental biology research field. BMC Dev. Biol.

[7] Ono M, Tsukamoto K, Tanimura N, Haritani M, Kimura K.M, Suzuki G, Okuda Y, Sato S (2004). An epizootic of subcutaneous tumors associated with subgroup a avian leukosis/sarcoma virus in young layer chickens. Avian. Dis.

[8] Venugopal K (1999). Avian leukosis virus subgroup J:A rapidly evolving group of oncogenic retroviruses. Res. Vet. Sci.

[9] Adkins H.B, Blacklow S.C, Young J.A (2001). Two functionally distinct forms of a retroviral receptor explain the nonreciprocal receptor interference among subgroups B, D, and E avian leukosis viruses. J. Virol.

[10] Aly M.M (2000). Isolation of a Subgroup J-like Avian Leukosis Virus Associated with Myeloid Leucosis in Meat Type Chickens in Egypt. Proceedings of the International Symposium on ALV-J and other Avian Retroviruses.

[11] Arafa A, Hussein H.A, Shalaby M.A, Aly M.M (2007). Serological and infection profiles of avian leukosis virus subgroup J in one day old chicks of broiler breeder chickens and their relatedness to virus vertical transmission. Egypt. J. Virol.

[12] Soliman M.A (2005). Immunological and Pathological Studies in Diagnosis of Viruses Induced Tumors (Avian Leukosis) in Chickens. Master's Thesis.

[13] Mousa S, Abdel-Wahab M.H (2009). Prevalence of avian leukosis virus in chicken flocks in upper Egypt. Assiut Vet. Med. J.

[14] Abdel Gayed M.B, Tamam S.M, Elkhawaga A.I, Hassan M.H (2017). Serological and Molecular Studies on Avian Leucosis Virus in Broiler Chicken in Egypt Viral Diseases 9/14/2017.

[15] Chai N, Bates P (2006). Na+/H+exchanger Type 1 is a receptor for pathogenic subgroup J avian leucosis virus. Proc. Natl. Acad. Sci. U. S. A.

[16] Chesters P.M, Smith L.P, Nair V (2006). E (XSR) element contributes to the oncogenicity of avian leukosis virus (subgroup J). J. Gen. Virol.

[17] Fadly A.M, Payne L.N, Saif Y.M, Barnes H.J, Fadly A.M, McDougald L.R, Swayne D.E, Glisson J.R (2002). Leukosis/sarcoma group. Diseases of Poultry.

[18] Payne L, Venugopal K (2000). Neoplastic disease:Marek's disease, avian leukosis and reticuloendotheleiosis. Rev. Sci. Tech.

[19] Holmen S.L, Federspiel M.J (2000). Selection of a subgroup a avian leukosis virus [ALV(A)] envelope resistant to soluble ALV(A) surface glycoprotein. Virology.

[20] Venugopal K, Smith L.M, Howes K, Payne L.N (1998). Antigenic variants of J subgroup avian leukosis virus:Sequence analysis reveals multiple changes in the Env gene. J. Gen. Virol.

[21] Davidson I (2001). Differential Diagnosis of Avian Oncogenic Viruses.

[22] Bancroft J.D, Suvarna K, Layton C (2012). Bancroft's Theory and Practice of Histological Techniques.

[23] Fenton S.P, Reddy M.R, Bagust T.J (2005). Single and concurrent avian leukosis virus infections with avian leukosis virus-J and avian leukosis virus-a in Australian meat-type chickens. Avian Pathol.

[24] Dorner A.J, Coffin J.M (1986). Determinants for receptor interaction and cell killing on the avian retrovirus glycoprotein *gp85*. Cell.

[25] Silva R.F, Fadly A.M, Taylor S.P (2007). Development of a polymerase chain reaction to differentiate avian leukosis virus (ALV) subgroups:Detection of an ALV contaminant in commercial Marek's disease vaccines. Avian Dis.

[26] Smith L.M, Brown S.R, Howes K, McLeod S, Arshad S.S, Barron G, Venugopal G, McKay J.C, Payne L.N (1998). Development and application of polymerase chain reaction (PCR) tests for the detection of subgroup J avian leukosis virus. Virus Res.

[27] Handberg K.J, Nielsen O.L, Jergensen P.H (2001). The use of serotype 1 and serotype 3 specific polymerase chain reaction for the detection of Marek's disease virus in chickens. Avian Pathol.

[28] Wei K, Sun Z, Zhu S, Guo W, Sheng P, Wang Z, Zhao C, Zhao Q, Zhu R (2012). Probable congenital transmission of reticuloendotheliosis virus caused by vaccination with contaminated vaccines. PLoS One.

[29] Bai J, Howes K, Payne L.N, Skinner M.A (1995). Sequence of host range determinants in the *Env* gene of a full-length, infectious proviral clone of exogenous avian leukosis virus HPRS-103 confirms that it represents a new subgroup (designated J). J. Gen. Virol.

[30] Kumar S, Stecher G, Tamura K (2016). MEGA7:Molecular evolutionary genetics analysis version 7.0 for bigger datasets. Mol. Biol. Evol.

[31] Schat K.A, Nair V, Saif Y.M, Fadly A.M, Glisson J.R, McDougald L.R, Nolan L.K, Swayne D.E Marek's disease. Diseases of Poultry.

[32] Abd-Ellatieff H.A, Abou Rawash A.A, Ellakany H.F, Goda W.M, Suzuki T, Yanai T (2017b). Molecular characterization and phylogenetic analysis of a virulent Marek's disease virus field strain in broiler chickens in Japan. Avian Pathol. J.

[33] Fadly A.M, Zavala G, Witter R.L, Saif Y.M, Fadly A.M, Glisson J.R, McDougald L.R, Nolan L.K, Swayne D.E (2008). Reticuloendotheliosis. Diseases of Poultry.

[34] Coffin J.M, Levy J.A (1992). Structure and classification of retroviruses. The Retroviridae. Vol. 1.

[35] Ewert D.L, DeBoer G.F, Perk K Avian lymphoid leukosis:Mechanism of lymphomagenesis. Immunodeficiency Disorders.

[36] Kilany W.H, Soliman M.A, Safwat M, Mehana O, El-Magid M.A, Marwa A.E, Hassan M.K, Nasif S.A (2015). Detection of avian leukosis virus subgroup J from commercial Peking duck breeder farm in Egypt. Int. J. Virol.

[37] Meng F, Li X, Fang J, Gao Y, Zhu L, Xing G, Tian F, Gao Y, Dong X, Chang S, Zhao P, Cui Z, Liu Z (2016). Genomic diversity of the Avian leukosis virus subgroup J *gp85* gene in different organs of an infected chicken. J. Vet. Sci.

[38] Mothes W, Boerger A.L, Narayan S, Cunningham J.M, Young J.A (2000). Retroviral entry mediated by receptor priming and low pH triggering of an envelope glycoprotein. Cell.

[39] Silva R.F, Fadly A.M, Hunt H.D (2000). Hypervariability in the envelope genes of subgroup J avian leukosis viruses obtained from different farms in the United States. Virology.

[40] Bova C.A, Manfredi J.P, Swanstrom R (1986). *Env* genes of avian retroviruses:Nucleotide sequence and molecular recombinants define host range determinants. Virology.

[41] Wang Z, Cui Z (2006). Evolution of *gp85* gene of subgroup J avian leukosis virus under the selective pressure of antibodies. Sci. China C Life Sci.

[42] Tsichlis P.N, Coffin J.M (1980). Recombinants between endogenous and exogenous avian tumor viruses:Role of the C region and other portions of the genome in the control of replication and transformation. J. Virol.

